# Evaluating age‐and gender‐related changes in brain volumes in normal adult using synthetic magnetic resonance imaging

**DOI:** 10.1002/brb3.3619

**Published:** 2024-07-05

**Authors:** Zuofeng Zheng, Yawen Liu, Zhenchang Wang, Hongxia Yin, Dongpo Zhang, Jiafei Yang

**Affiliations:** ^1^ Department of Radiology Beijing ChuiYangLiu Hospital Beijing China; ^2^ Department of Radiology Beijing Friendship Hospital, Capital Medical University Beijing China

**Keywords:** aging, brain segmentation, quantitative MRI, synthetic MRI

## Abstract

**Objective:**

Normal aging is associated with brain volume change, and brain segmentation can be performed within an acceptable scan time using synthetic magnetic resonance imaging (MRI). This study aimed to investigate the brain volume changes in healthy adult according to age and gender, and provide age‐ and gender‐specific reference values using synthetic MRI.

**Methods:**

A total of 300 healthy adults (141 males, median age 48; 159 females, median age 50) were underwent synthetic MRI on 3.0 T. Brain parenchymal volume (BPV), gray matter volume (GMV), white matter volume (WMV), myelin volume (MYV), and cerebrospinal fluid volume (CSFV) were calculated using synthetic MRI software. These volumes were normalized by intracranial volume to normalized GMV (nGMV), normalized WMV (nWMV), normalized MYV (nMYV), normalized BPV (nBPV), and normalized CSFV (nCSFV). The normalized brain volumes were plotted against age in both males and females, and a curve fitting model that best explained the age dependence of brain volume was identified. The normalized brain volumes were compared between different age and gender groups.

**Results:**

The approximate curves of nGMV, nWMV, nCSFV, nBPV, and nMYV were best fitted by quadratic curves. The nBPV decreased monotonously through all ages in both males and females, while the changes of nCSFV showed the opposite trend. The nWMV and nMYV in both males and females increased gradually and then decrease with age. In early adulthood (20s), nWMV and nMYV in males were lower and peaked later than that in females (*p* < .005). The nGMV in both males and females decreased in the early adulthood until the 30s and then remains stable. A significant decline in nWMV, nBPV, and nMYV was noted in the 60s (Turkey test, *p* < .05).

**Conclusions:**

Our study provides age‐ and gender‐specific reference values of brain volumes using synthetic MRI, which could be objective tools for discriminating brain disorders from healthy brains

## Graphical abstract

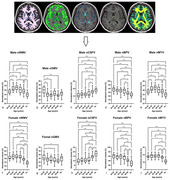

## 1 INTRODUCTION

Normal brain aging is a complex process that occurs in absence of concurrent pathology, and brain volume estimation is important for understanding this process. A variety of neurodegenerative diseases, such as multiple sclerosis (Ghione et al., 2019; Moridi et al., 2022), Alzheimer's disease (Boublay et al., 2020), and vascular dementia (Li et al., 2017), can accelerate the process of brain atrophy. Although quantification of brain volume may be useful for early diagnosis and management of these brain disorders, it is still challenging to determine whether brain atrophy is caused by normal brain aging or pathological conditions. Therefore, quantifying the specific patterns of age‐ and gender‐related brain volume changes is crucial for neuroscientific research and clinical diagnosis.

Magnetic resonance imaging (MRI) is a noninvasive technique, which provides a useful tool to evaluate the human brain volume in vivo (Buchpiguel et al., 2020; Good et al., 2001; Oschwald et al., 2019). Currently, the most commonly used brain segmentation methods rely on the signal intensity, which is based on the conventional contrast‐weighted MR images and related offline methods, such as statistical parametric mapping (SPM) (Serai et al., 2019), FMRIB Software Library (FSL) (Narayanan et al., 2020), and FreeSurfer (Guo et al., 2019; Kijonka et al., 2020). However, these methods involve complex postprocessing steps and are relatively time consuming. Furthermore, acquisition parameters and scanner settings can potentially influence the signal intensities of conventional contrast‐weighted images. These drawbacks limit their wide use in routine clinical practice.

Recently, brain segmentation and volume estimation can be performed based on quantitative values, such as T1, T2 relaxation times and proton density (PD), by the synthetic MRI (SyMRI) method, and multi‐dynamic multi‐echo (MDME) sequence is widely used for SyMRI in clinical practice (Hagiwara et al., 2017). These quantitative parameters represent physical constants that are presumably intrinsic to a given tissue or other material. Fully automatic brain tissue segmentation and volumetry has been implemented in SyMRI software with postprocessing time <1 min, which is much more convenient in clinical practice. Previous studies showed that gray matter (GM) and white matter (WM) volume estimation agree well with SPM analyses (Serai et al., 2019), and the repeat measurement errors for brain parenchymal volume (BPV), intracranial volume (ICV), brain parenchymal fraction (BPF), and GM fraction measured by MDME method at 3.0 T were significantly lower than those measured by FreeSurfer, FSL, or SPM (Granberg et al., 2016). In previous studies, the age‐related changes in brain tissue volumes have been evaluated using MDME sequence (Hagiwara et al., 2021; Lee et al., 2018). However, the sample sizes in these studies were relatively small and gender‐related brain volume changes were not evaluated based on MDME sequence.

Hence, the aim of this study was to investigate the age‐ and gender‐related brain volume changes in healthy adults based on SyMRI and to provide reference values derived from MDME sequence on 3.0 T MRI scanner.

## 2 MATERIALS AND METHODS

### 2.1 Subjects

This study was approved by our institutional review board, and the informed consents were written by all the participants. Inclusion criteria were as follows: (1) no history of a major medical condition and no previous diagnosis of neurological or psychiatric disorder; (2) age older than 20 years; (3) no contraindications to brain MRI. The exclusion criteria include (1) white matter hyperintensity (WMH) on fluid‐attenuated inversion recovery (FLAIR) with Fazekas scale (Fazekas et al., 1993) of 2 or higher; (2) other brain abnormalities detected on all the MRI sequences, such as infarcts, microbleeds, old hemorrhage, and intracranial mass, and (3) obvious MRI artifact preventing image analysis. Images were analyzed for artifacts by one author (8 years of neuroimaging experience) in consultation with another author (10 years of neuroimaging experience). Based on the criteria, 10 volunteers with WMH Fazekas scale of 2 or higher, 2 with obvious motion artifact, 1 with meningioma, 2 with chronic lacuna infarct, 1 with traumatic brain injury were excluded from the final analysis. Finally, 300 subjects (141 males: age range 22–81, median age 48 years; 159 females: age range 21–85, median age 50 years) were included in this study. The details of age distribution are shown in Table 1.

**TABLE 1:** The characteristics of subjects in six age groups.

Age group(years)	20–29	30–39	40–49	50–59	60–69	70–
Males(*n* = 141)	19	26	28	22	27	19
Median age (years)	27.0	35.0	45.0	56.0	63.0	73.0
Females(*n* = 159)	27	27	24	31	30	20
Median age (years)	26.0	34.0	43.0	55.0	65.0	76.5

### 2.2 MRI acquisition

All MRI examinations were performed on a 3.0 T MRI scanner (SIGNA Pioneer; GE Healthcare) using a 32‐channel head coil. Quantitative MRI was performed using MDME sequence. This sequence is a multisection, multiecho, multisaturation delay method of saturation recovery acquisition that uses a fast spin‐echo readout (J. B. M. Warntjes et al., 2008). Two echo times (TE) and four delay times (TD) were used to quantify longitudinal T1 and transverse T2 relaxation times, and eight complex images per slice were produced. To retrieve T1, T2, and PD maps, while accounting for B1 inhomogeneity, a least square fit was performed on the signal intensity (I) of images by minimizing the following equation:

$$\begin{array}{ccc} I & = & \;A.\mathrm{PD}.\exp\left(-TE/T2\right)\\ & & \;\;\frac{1-\left\{1-\cos\left(B_{1}\theta \right)\right\}\exp\left(-TI/T1\right)-\cos\left(B_{1}\theta \right)\exp\left(-TR/T1\right)}{1-\cos\left(B_{1}\alpha \right)\cos\left(B_{1}\theta \right)\exp\left(-TR/T1\right)} \end{array}$$

where *α* is the applied excitation flip angle (90°) and *θ* is the saturation flip angle (120°). A is an overall intensity scaling factor that takes into account several elements, including sensitivity of the coil, amplification of the radiofrequency chain, and voxel volume.

The parameters used for MDME sequence were as follows: repetition time (TR), 4341 ms; TE1/TE2, 21 ms/87 ms; echo train length, 12; matrix, 320 × 256; parallel imaging acceleration factor, 2; field of view, 240×240; slice thickness/interslice gap, 4/1 mm; section, 30. The acquisition time was 4:55 min.

### 2.3 Image postprocessing by synthetic MRI

The method of brain tissue segmentation by SyMRI has been described in detail in previous study (Hagiwara et al., 2017). The measured T1, T2, and PD values of brain tissues can be used as coordinates in a R1‐R2‐PD space. The previously reported quantitative values for WM, GM, and cerebrospinal fluid (CSF) for healthy controls derived from SynMRI were used as reference values to define each brain tissue (J. B. M. Warntjes et al., 2008). A numerical block simulation was performed to investigate R1, R2, and PD for tissue mixtures and their ratios (J. West et al., 2012). Using this method, the tissue fractions in each voxel can be calculated, and the fractions change in 0.1% increments from 0 to 100. Voxels that were not categorized as WM, GM, or CSF or mixtures of these tissues were termed non‐WM/GM/CSF (NoN). The BPV was calculated as the sum of the volumes of WM, GM, and NoN. The border of the ICV was defined at a PD of 50%, assuming that the border of ICV corresponds to the interface between CSF (PD = 100%) and bone (PD = 0%) (Ambarki et al., 2012), and the ICV was calculated as the sum of BPV and volume of CSF.

The myelin volume (MYV) in each voxel was estimated based on a 4‐compartment model (the MY partial volume, the cellular partial volume, the free water partial volume, and the excess parenchymal water partial volume). This model postulates that the 4 compartments have their own R1, R2, and PD values and contribute to the effective R1, R2, and PD values in an acquisition voxel. The partial volume fractions of the four compartments were estimated by performing Bloch equations, and the MYV was calculated by multiplying the myelin volume fraction (MYF) by the volume of each voxel (M. Warntjes et al., 2016).

**FIGURE 1:**
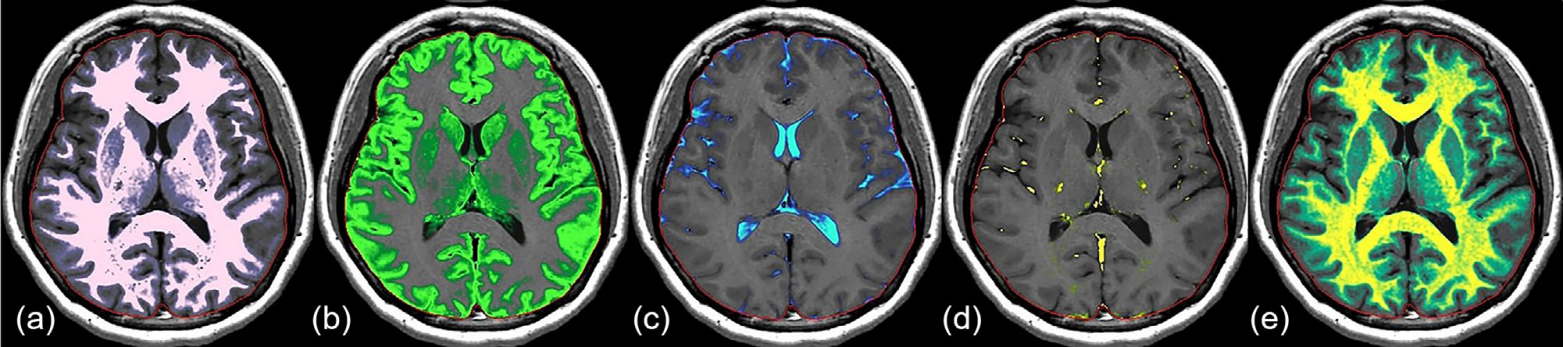
An example of brain tissue segmentation using synthetic magnetic resonance imaging (MRI). white matter (WM), (a), gray matter (GM) (b), cerebrospinal fluid volume (CSF) (c), non‐WM/GM/CSF (NoN) (d), and myelin (MY) (e) maps are overlaid on a synthetic T1‐weighted image (TR/TE = 500/10 msec).

The acquired raw DICOM data of all the subjects were processed using SyMRI software (SyntheticMR AB, version 8.0.4), and the segmented brain tissue volumes and MYF can be obtained automatically (Figure 1). The total processing time for each subject was <1 min.

### 2.4 Brain volume normalization

We use the proportions method to normalize brain volumes in order to deal with inter‐individual variability in total head size (Buchpiguel et al., 2020; Hagiwara et al., 2021). The normalized brain volume was calculated as the volume of brain tissues divided by ICV. Finally, we obtained the normalized WMV (nWMV), normalized GM volume (nGMV), normalized CSF volume (nCSFV), normalized BPV (nBPV), and normalized MYV (nMYV).

### 2.5 Statistical analysis

The subjects were stratified into each decade, and totally six age groups (20–29, 30–39, 40–49, 50–59, 60–69, ≥70) were obtained for male or female participants (Table 1). Kolmogorov–Smirnov test was used to evaluate the normality of continuous data. Continuous data were expressed as mean ± standard deviation (SD). To verify the validity of adjusting each segmented brain tissue volumes by ICV, the correlation analysis was performed between BPV and ICV, and one‐way analysis of variance was performed to evaluate the differences of ICV between six age groups. We compare the age, overall segmented brain tissue volumes, and normalized brain tissue volumes between males and females using independent sample *t* test or Mann–Whitney *U* test based on the data distribution and homoscedasticity. The differences of normalized brain tissue volumes between six different age groups were assessed using one‐way analysis of variance or Kruskal–Wallis test. In case, a significant effect was revealed, a post Turkey's multiple comparisons test or Dunn's multiple comparisons test was performed. To investigate the relationship between age and normalized brain tissue volumes, regression analysis was performed as function of age separated by sex, and the correlation coefficient (*R*) was calculated. We selected quadratic or linear approximation by choosing the one that showed the higher correlation coefficient. The statistical analysis was performed using GraphPad Prism 9.0 and SigmaPlot 14.0. The level of statistical significance was set at *p* < .05.

## 3 RESULTS

Pearson correlation analysis showed that significant strong correlations were revealed between ICV and BPV in both males and females (males: *R* = 0.852, *p* < .001; females: *R* = 0.823, *p* < .001) and no statistical difference of ICV was revealed between different age groups in both males and females (males: *p* = .3462; females: *p* = .1606). Therefore, it was necessary and appropriate to normalize the brain tissue volumes by ICV to minimize the inter‐individual variability in total head size.

Table 2 shows the comparison results of overall segmented brain tissue volumes and normalized brain tissue volumes between males and females. There was no statistical difference in age between males and females (*p* = .784). The segmented brain volumes were significantly larger in males than in females (*p* < .05). After normalization by ICV, nGMV and nBPV were significantly smaller in males than in females (*p* = .010, *p* < .001, respectively), and nCSFV was significantly larger in males than in females (*p* = .001), where there were no significant differences in nWMV and nMYV between the two gender groups (*p* = .054 and .057, respectively).

**TABLE 2:** Comparisons of age and measured brain tissue volumes between males and females.

Median age, (range), years	Males(*n* = 141)	Females(*n* = 159)	*p*
	48.0(22–81)	50.0(21–85)	.784
Brain volume(mL)			
WMV	597.0 ± 73.7	539.2 ± 66.7	<.001
GMV	640.2 ± 69.0	580.2 ± 52.3	<.001
CSFV	209.1 ± 65.0	163.6 ± 51.2	<.001
MYV	196.8 ± 29.3	178.6 ± 27.4	<.001
BPV	1294 ± 107	1163 ± 86	<.001
ICV	1503 ± 125	1326 ± 86	<.001
Normalized brain volume(%)			
nWMV	39.7 ± 3.9	40.6 ± 4.0	.054
nGMV	42.7 ± 4.1	43.8 ± 3.8	.010
nCSFV	13.8 ± 3.8	12.3 ± 3.7	.001
nBPV	86.1 ± 3.8	87.7 ± 3.7	<.001
nMYV	13.1 ± 1.6	13.4 ± 1.7	.057

*Note*: *p* values are for comparisons between males and females. The comparisons of CSFV, nGMV, nCSFV, nBPV, and age were performed with Mann–Whitney *U* test, and comparisons of other brain tissue volumes were analyzed using independent sample *t* test. Differences were considered significant at *p* < .05 (two‐tailed). The measured brain tissue volumes data were presented as means ± standard deviations.

Abbreviations: BPV, brain parenchymal volume; CSFV, cerebrospinal fluid volume; GMV, gray matter volume; ICV, intracranial volume; MYV, myelin volume; nBPV, normalized brain parenchymal volume; nCSFV, normalized cerebrospinal fluid volume; nGMV, normalized gray matter volume; nMYV, normalized myelin volume; nWMV, normalized white matter volume; WMV, white matter volume.

Table 3 shows the comparison results of normalized brain tissue volumes between males and females within the same age groups. In 20–29 and 50–50 age groups, nWMV and nMYV were significantly larger in females (nWMV: *p* < .001, *p* = .035, respectively; nMYV: *p* = .001, *p* = .015, respectively). In 40–49 and 60–69 age groups, nGMV was significantly larger in females (*p* = .005, *p* = .032, respectively). In 30–39 and 40–49 age groups, nBPV was significantly larger in females (*p* = .008, *p* = .023, respectively) and nCSFV was significantly lower in females (*p* = .005, *p* = .028, respectively).

**TABLE 3:** Comparisons of normalized brain volumes between males and females in different age groups.

Age (years)	nWMV(%)		nGMV(%)		nCSFV(%)		nBPV(%)		nMYV(%)	
	Males	Females	Males	Females	Males	Females	Males	Females	Males	Females
20–29	38.0 ± 2.4	41.3 ± 2.6^****^	47.4 ± 4.6	45.9 ± 4.2	11.0 ± 3.1	10.0 ± 3.3	89.0 ± 3.1	90.0 ± 3.3	12.4 ± 1.3	13.7 ± 1.1^***^
30–39	41.5 ± 3.0	43.1 ± 3.3	41.9 ± 3.8	43.7 ± 3.3	12.4 ± 2.6	10.5 ± 2.1^**^	87.6 ± 2.6	89.5 ± 2.1^**^	14.1 ± 1.2	14.2 ± 1.5
40–49	42.7 ± 2.9	42.5 ± 3.4	41.2 ± 2.5	43.6 ± 3.2^**^	12.7 ± 3.0	10.9 ± 2.9^*^	87.2 ± 3.1	89.1 ± 2.8^*^	14.1 ± 1.0	14.2 ± 1.3
50–59	40.1 ± 3.6	42.4 ± 3.1^*^	43.2 ± 4.0	42.6 ± 3.7	12.8 ± 3.2	11.5 ± 2.9	87.2 ± 3.3	88.6 ± 2.9	13.2 ± 1.3	14.2 ± 1.4^*^
60–69	39.0 ± 3.3	38.8 ± 2.4	40.8 ± 3.0	42.6 ± 3.2^*^	16.0 ± 3.3	14.6 ± 2.4	84.0 ± 3.3	85.4 ± 2.4	12.9 ± 1.4	13.1 ± 1.0
≥70	34.9 ± 2.5	34.3 ± 2.5	43.2 ± 3.5	45.0 ± 3.9	18.3 ± 2.8	17.4 ± 3.3	81.8 ± 2.8	82.6 ± 3.3	10.9 ± 1.3	10.7 ± 1.3

*Note*: The comparisons of normalized brain tissue volumes between males and females were performed by independent sample *t* test or Mann–Whitney *U* test. Differences were considered significant at *p* < .05 (two‐tailed). * Value for statistically different compared to male groups: *p < .05, **p < .01, ***p < .005, ****p < .001. Data were presented as means ± standard deviations.

Abbreviations: nBPV, normalized brain parenchymal volume; nCSFV, normalized cerebrospinal fluid volume; nGMV, normalized gray matter volume; nMYV, normalized myelin volume; nWMV, normalized white matter volume.

**FIGURE 2:**
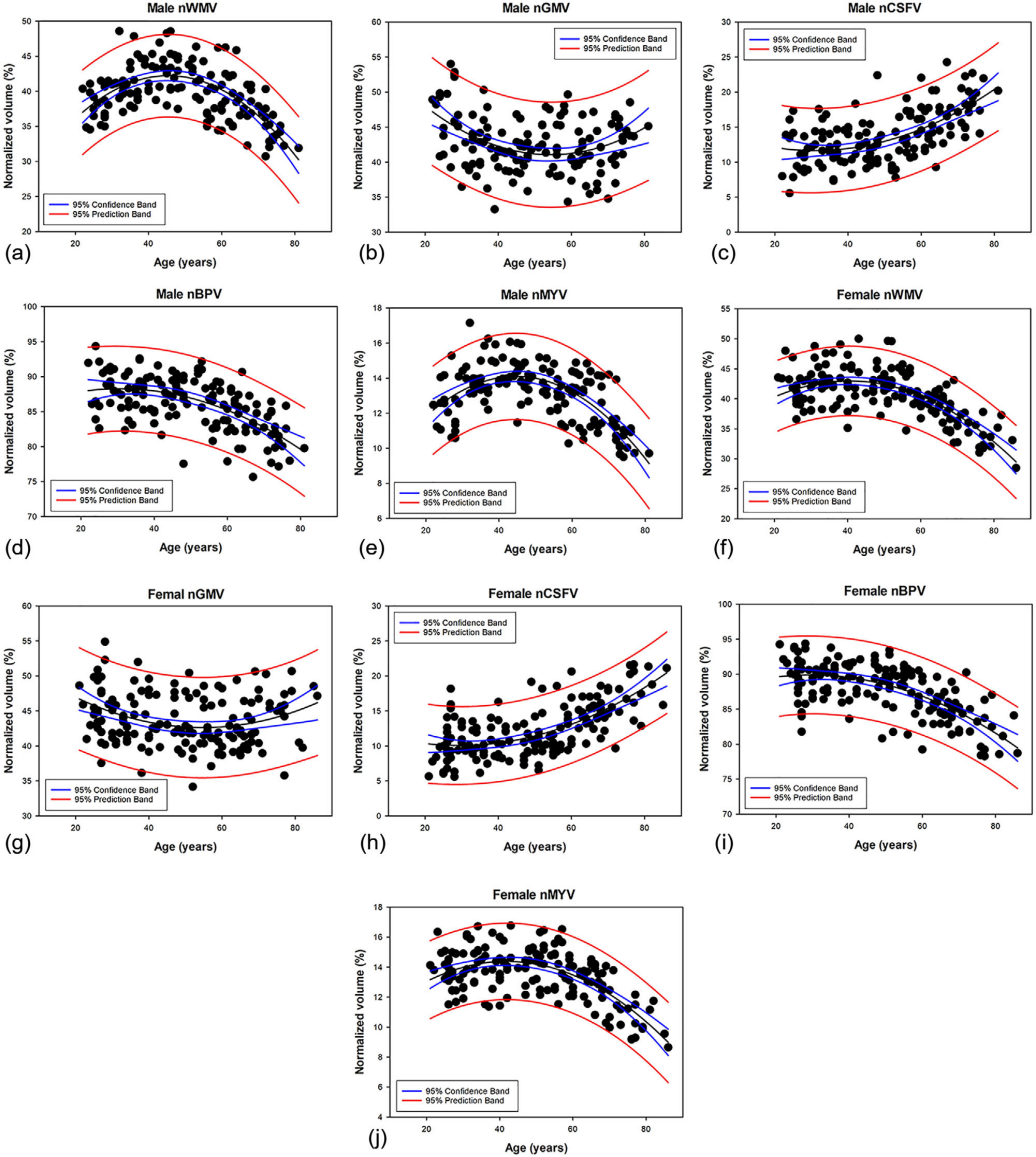
Scatterplots and approximate curves of normalized white matter volume (nWMV), normalized gray matter volume (nGMV), normalized cerebrospinal fluid volume (nCSFV), normalized brain parenchymal volume (nBPV), and normalized myelin volume (nMYV) in relation to age in both males (a–e) and females (f–j). Regression lines (black lines) are shown with 95% confidence intervals (blue lines) and 95% prediction intervals (red lines).

**FIGURE 3:**
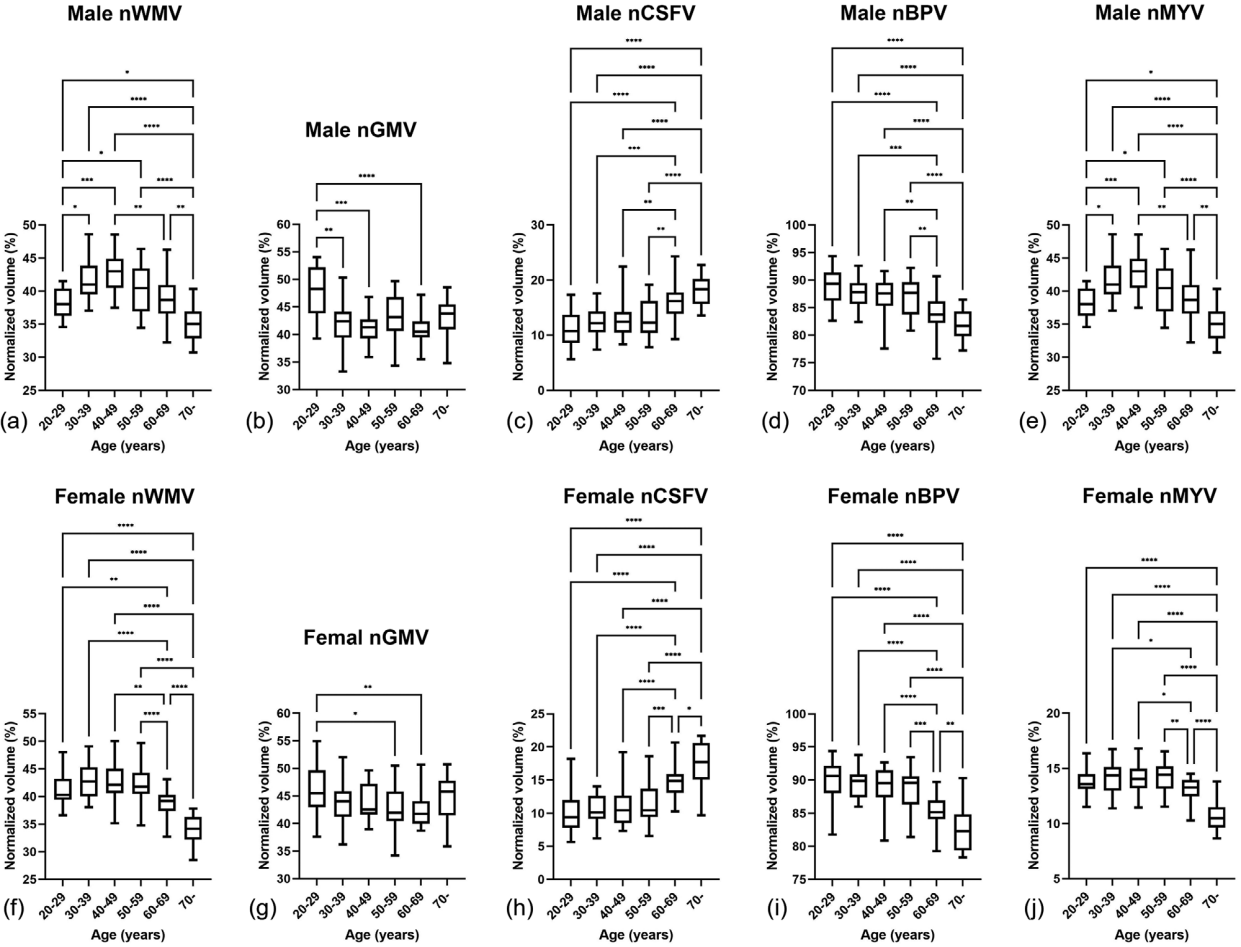
Statistical results of normalized white matter volume (nWMV), normalized gray matter volume (nGMV), normalized cerebrospinal fluid volume (nCSFV), normalized brain parenchymal volume (nBPV), and normalized myelin volume (nMYV) between different age groups in both males (a–e) and females (f–j). The comparisons of nGMV were performed with Kruskal–Wallis test in both gender groups, and comparisons of nWMV, nCSFV, nCSFV, nBPV were analyzed using one‐way analysis of variance. (*p < .05, **p < .01, ***p < .005, ****p < .001).

The changes of normalized brain tissue volumes in relation to age are shown in Figure 2. The approximate curves of nGMV, nWMV, nCSFV, nBPV, and nMYV were best fitted by quadratic curves. Table 4 shows the equations used to plot the nGMV, nWMV, nCSFV, nBPV, and nMYV curves in males and females. The correlation coefficients (*R*) were >0.6 for all the estimations, except for nGMV (males, *R* = 0.3857; females, *R* = 0.2950). Figure 3 shows the comparison results of normalized brain tissue volumes between six age groups in both males and females. The nBPV decreased monotonously through all ages in both males and females, and it decreased faster after around 60s, while the changes of nCSFV showed the opposite trend. Multiple comparisons test for nBPV and nCSFV did not show significant differences among younger groups (<60 years), whereas it showed significant differences between younger groups (<60 years) and the older groups (≥60 years). The nWMV and nMYV in both males and females seemed to increase gradually and then decrease with age. In males, nWMV and nMYV increased more rapidly until the 40s, and they declined thereafter. While in females, they reached a peak at the age of 30s, and gradually decreased around 60s, then decreased faster thereafter. Multiple comparison test for nWMV and nMYV did not show significant differences among younger females (60 years), whereas they showed significant differences between younger females (<60 years) and the older ones (≥60 years). The nGMV in both males and females decreased in the early adulthood until the 30s and then remains stable.

**TABLE 4:** The correlation between normalized brain volumes and age.

	Normalized volume (%)	First coefficient		Second coefficient		Third coefficient		*R*
		SD	*p*	SD	*p*	SD	*p*	
Males								
nWMV	22.6 + 0.420 × age − 0.0095 × age^2^	2.621	<.0001	0.1121	<.0001	0.0011	<.0001	0.6482
nGMV	58.4 − 0.638 × age + 0.0059 × age^2^	3.335	<.0001	0.1426	<.0001	0.0014	<.0001	0.3857
nCSFV	15.1 − 0.224 × age + 0.0036 × age^2^	2.667	<.0001	0.1140	.0519	0.0011	.0017	0.6116
nBPV	85.0 + 0.211 × age − 0.0036 × age^2^	2.686	<.0001	0.1148	.0597	0.0011	.0022	0.6064
nMYV	6.63 + 0.335 × age − 0.0038 × age^2^	1.093	<.0001	0.0467	<.0001	0.0005	<.0001	0.6488
Females								
nWMV	32.5 + 0.523 × age − 0.0065 × age^2^	2.057	<.001	0.0877	<.001	0.0009	<.001	0.6972
nGMV	53.4 − 0.394 × age + 0.0036 × age^2^	2.547	<.001	0.1086	.0004	0.0010	.0009	0.2950
nCSFV	12.9 − 0.193 × age + 0.0032 × age^2^	1.967	<.001	0.0839	.0239	0.0008	<.001	0.6666
nBPV	87.1 + 0.192 × age − 0.0033×age^2^	1.967	<.001	0.0839	.0235	0.0008	<.001	0.6673
nMYV	9.51 + 0.233 × age − 0.0028×age^2^	0.9048	<.001	0.0386	<.001	0.0004	<.001	0.6520

*Note*: The first coefficient is the *Y*‐intercept; the second coefficient is for age; and the third coefficient is for age^2^. *p* values represent the statistical results of the coefficients. *R* values represent the correlation coefficients.

Abbreviations: nBPV, normalized brain parenchymal volume; nCSFV, normalized cerebrospinal fluid volume; nGMV, normalized gray matter volume; nMYV, normalized myelin volume; nWMV, normalized white matter volume; SD, SD, standard deviation.

## 4 DISCUSSION

In this study, we evaluated the changes of brain tissue volumes in healthy adults associated with aging based on SyMRI on 3.0 T MRI scanner. Automatic brain segmentation and volume estimation were preformed using synthetic MR software in <1 min. The changes of nGMV, nWMV, nCSFV, nBPV, and nMYV in relation to age were better fitted by quadratic curves. The nBPV decreased monotonously through all ages in both males and females, while the changes of nCSFV showed the opposite trend. The nWMV and nMYV in both males and females seemed to increase gradually and then decrease with age, while nGMV decreased in the early adulthood until the 30s and then remains stable. In early adulthood (20s), the nWMV and nMYV in males were lower and peaked later than that in females. The nWMV, nMYV, and nBPV decreased faster after around 60s. Our results were similar with the previous studies (Hagiwara et al., 2021; Scahill et al., 2003; Vågberg et al., 2016).

Several brain segmentation methods have been used for brain volume estimation, most of which are performed based on differences in contrast between different brain tissues (Fletcher et al., 2018; Serru et al., 2021). Although conventional images provide relatively good anatomic detail, the signal intensity is not absolute, and the post‐processing process is complex, including filters and normalization of signal intensities (Serai et al., 2019). SyMRI can result in absolute values for all brain tissue properties, which are independent of imperfections intrinsic to the MRI scanners or variations in the pulse sequence (J. B. M. Warntjes et al., 2008). Conventional segmentation method assumes that each voxel is assigned to one specific tissue type, which may cause partial volume effect. SyMRI method calculates brain tissue fractions in each voxel, allowing more than one tissues in one voxel, which can remove the dependence on spatial resolution and decrease the scan time required for high resolution (Hagiwara et al., 2017). Fully automatic brain tissue segmentation and volumetry is now implemented in SyMRI software, which can be launched in the PACS system, making it more convenient in clinical practice.

In our study, the segmented brain volumes were significantly larger in males than that in females, in line with the previous studies (Courchesne et al., 2000; Hagiwara et al., 2021). This is mainly related to the fact that males have larger ICV, while BPV is dependent on the skull size. Our study showed that BPV had a strong linear correlation with ICV, indicating that it is appropriate to normalize the brain tissue volumes using ICV to minimize the inter‐individual variability in total head size. In previous studies, it was generally accepted that BPV decreases gradually with age (Courchesne et al., 2000; Hagiwara et al., 2021). We demonstrated that nBPV decreased monotonously through all ages in both male and female adults, and the inverted U‐shaped quadratic curve was better fitted than a line as a function of age, which was in line with the previous studies (Hagiwara et al., 2021; Vågberg et al., 2016). Our results showed that the nBPV decreased slowly until around the 60s, and then it decreased faster after 60s in both males and females. However, Hagiwara et al. (2021) study showed that the age of rapid decline in brain parenchymal fraction was around 50s. The observed differences may partly be related to the sample sizes and age differences in different age groups.

Our study demonstrated that the inverted U‐shapes were shown by both nWMV and nMYV, with peaks at about 30s to 40s, and the volumes reduction accelerated after the age of 60. Previous studies also reported that nWMV increased until around 40s, and inverted U‐shape was also revealed (Bethlehem et al., 2022). The gradual increase in nWMV and nMYV in the early adulthood reflected the process of myelination, which has been shown by histology of human brains (Benes, 1989). WMV reflects the total volume of myelinated axons, while MYV only represents the total volume of MY, excluding the axons, extracellular water and other materials that are not MY (McAllister et al., 2017). MYV can be evaluated by many MRI methods, such as magnetization transfer imaging (K. L. West et al., 2018), measurement of multiexponential T2 relaxation time (Alonso‐Ortiz et al., 2015), or the ratio of T1‐weighted and T2‐weighted images (Glasser & Essen, 2011). Limitations of these methods are mainly practical and it is difficult to evaluate MYV using conventional brain segmentation methods such as SPM, and FreeSurfer. Using SyMRI method, the content of MY and edema can be easily estimated based on the 4‐compartment model in routine clinical practice (M. Warntjes et al., 2016). However, a very limited study has compared the MYV measurement by SyMRI with other methods or histology, and the validation research is still needed. Our study showed that the nWMV and nMYV in males were lower in younger group (20s) and peaked later than that in females. Previous study also showed that brain volumetric differences between sexes persist throughout the aging process (Buchpiguel et al., 2020). The reason for this difference between the genders is not clear and may be related to the role of sex hormones (Eberling et al., 2004).

It is generally accepted that aging is associated with gray matter volume (GMV) reduction in healthy adults (Hagiwara et al., 2021; Pfefferbaum et al., 1994), although the reduction pattern varies in different studies. Our study showed that the change of nGMV was better fitted by quadratic curves, which was consistent with previous study (Hagiwara et al., 2021) and other studies reported a linear decrease pattern in nGMV (Farokhian et al., 2017; Taki et al., 2011). The observed differences across studies might be due to several reasons, including MRI segmentation methods, normalization approaches of brain tissue volumes (Voevodskaya et al., 2014), and sample sizes in different age groups. Conventionally, T1‐weighted images are the most commonly used structure MRI sequence for segmentation and estimation of brain volumes, and WMHs are a very common finding on healthy aging brain, with intensities that can be very similar to cortical and subcortical gray matter (Dadar et al., 2017). Previous study showed that presence of WMHs can lead to systematic inaccuracies in GM segmentations based on FreeSurfer, which can also change the associations between GMV and clinical outcomes (Dadar et al., 2021). Unlike the conventional segmentation methods, SyMRI allows for automated segmentation based on the quantitative T1, T2, and PD values of GM, WM, and CSF measured by SyMRI for healthy controls (J. B. M. Warntjes et al., 2008). McAllister et al.’s study showed that the GMV began to decrease even at a younger age around 6–7 years of age, after an increase during the developmental period (McAllister et al., 2017). In our study, contrary to the nWMV and nMYV, the nGMV in both males and females decreased in the early adulthood until the 30s and then remains stable. However, Hagiwara et al.’ s study showed that GM fraction remains stable around the 60s (Hagiwara et al., 2021). The differences may be related to the sample sizes in different age groups. In Hagiwara's study, the median age of subjects was 66.5 years, which is much older than that in ours. The larger density of subjects in old age groups may introduce some bias in the results.

In this study, we only focus on the age‐related volumetric data; various contrast‐weighted images and quantitative relaxation data can also be created based on SyMRI. Recently, 3D quantitative SyMRI has been used in clinical research, providing a relatively high resolution in the section direction, and the total scan time was about 9 min, which was much shorter than the conventional sequences (Fujita et al., 2021). Advances in machine learning have also been applied to various part of SyMRI work flow. Using advanced reconstruction method, the effective signal to noise ratio efficiency can be increased and the scan time can be reduced with fast SyMRI sequence, which is practical for motion‐prone patients (Hwang & Fujita, 2022). Regarding future perspectives, the combination of multiparametric quantitative and qualitative information can provide much more valuable information for the diagnosis and management of brain diseases.

There are some limitations in our study. First, our study is a cross‐sectional design. Longitudinal study is superior, which can reduce the inter‐subject variance. Second, although we excluded the subjects with major medical condition, neurological, or psychiatric disorder, we did not consider the life background of the subjects, including hypertension, race, drinking, smoking, and occupation. Further research is needed to evaluate the age‐related changes in brain volume, considering the effects of these factors.

## 5 CONCLUSION

In conclusion, we obtain the age‐ and gender‐related quantitative volume data of healthy adult brain using SyMRI technique, which was rapid and easily translatable to clinical practice. Even though the overall changes of normalized brain volumes in relation to age were similar between males and females, certain differences were found between them, indicating the importance of matching gender and age in analyzing disorders. Our results may be useful for discriminating brain diseases from healthy brains using SyMRI.

## AUTHOR CONTRIBUTIONS

**Zuofeng Zheng**: Conceptualization; data curation; formal analysis; methodology; writing—original draft. **Yawen Liu**: Conceptualization; data curation; formal analysis; methodology; software; writing—original draft. **Zhenchang Wang**: Conceptualization; methodology; writing—review and editing. **Hongxia Yin**: Conceptualization; methodology; project administration; writing—review and editing. **Dongpo Zhang**: Data curation; writing—review and editing. **Jiafei Yang**: Data curation; writing—review and editing.

## CONFLICT OF INTEREST STATEMENT

The authors declare no conflicts of interest.

### PEER REVIEW

The peer review history for this article is available at https://publons.com/publon/10.1002/brb3.3619

## Data Availability

Data available on request from the authors.
